# The Temperature in Shock

**Published:** 1904-01-23

**Authors:** 


### THE TEMPERATURE IN SHOCK.

In an admirable thesis, for which he was awarded the Fellowship in Surgery at Rush Medical College, Dr. G. C. Kinnaman[1] sets forth the results of an experimental research into the temperature relationship existing in shock. His experiments were carried out upon dogs. In every case ether was used as the anæsthetic, and observations were taken during each experiment of the rectal temperature, the blood-pressure and the frequency of respiratory movements. The blood-pressure tracing was recorded by means of connecting a cannula in the right carotid artery with a mercurial manometer. The respirations were taken by means of encircling the lower part of the chest with an elastic band, and inserting a small bag between the band and the chest; the bag was connected by rubber tubing with a tambour attached to a kymograph. The temperature was taken by placing a thermometer in the rectum. Three series of experiments were carried out. In series A the abdomen was opened and the intestines exposed and spread out upon the abdomen. No manipulation was practised upon them. In this series the shock which was produced was entirely dependent upon the simple exposure of the intestines. In every case there was a gradual and uniform drop in temperature from the time the intestines were exposed until death resulted from shock. The curve of fall in every case was regular, and there was no acceleration or retardation in any part. The fall ranged from 3 8° C. to 6·2° C., the average being 5·7° C. The time required to produce death varied from 1·46 to 3·51 hours, giving an average of 2 44. In all cases there was a marked fall in blood-pressure, but the descent was neither regular nor continuous.

In series B, the intestines were exposed and spread upon the abdomen. The dog was then immediately immersed in a bath of normal saline solution which was kept at a temperature as near as possible to the normal level for the dog, the head only of the animal being kept above the surface of the solution. In these experiments the bath had a marked influence in preventing the fall in temperature, although in every case there was a slight fall, averaging 1·25° C. below the temperature of the bath. A wide variation was shown in the blood-pressure fall, the maximum being 89 m.m., and the minimum 18 m.m. In every case there was marked acceleration of respiration. In none of the cases did death ensue from shock. In series C, the abdomen was opened and the intestines exposed as in the previous experiments. They were then vigorously manipulated almost continuously until the production of presumably fatal shock. At this point the dog was transferred to the saline bath, which was kept at a temperature slightly higher than the normal temperature of the dog. Manipulation was ceased and the effect of the bath noticed. Here there was a gradual and uniform fall in the animal's temperature from the time the intestines were exposed until the production of the presumably fatal shock. At no time was there a demonstrable tendency to acceleration or retardation. The average fall was 5·77° C. The average normal temperature was 38·9° C., and that at which shock was considered fatal was 33·1° C. After transferring the animal to the warm bath a very marked effect was produced in raising its temperature. The average rise was 3 8° C. in an average time of forty-seven minutes. In every case there was a decided though not continuous fall of blood-pressure from the commencement of the experiment up to the time of transference to the warm bath. From this point a consistent and progressive though irregular rise of temperature took place. Previous to immersion in the bath the respirations were considerably slowed in three out of the four cases, with an average of 17 to the minute. In the fourth case there was an increase of 4 per minute in the rate. In every instance a great variance in the rate was evident from time to time. After transference to the bath marked acceleration, 15 per minute, was shown, while in the fourth case, after an acceleration of 6 per minute the rate fell to 3 below the shock-rate.

From these experiments Dr. Kinnaman concludes that shock must not be considered as due to the lowering or exhaustion of one bodily function, but as a composite condition embracing a lowering of the blood-pressure, an interference with the respiratory act, and a marked fall in the body temperature, the last mentioned being the most uniform and progressive factor. Moreover, if the fall of temperature is prevented, there will not be a fall of blood-pressure or slowing of respiration, and when shock has already occurred, the symptoms will pass off as the temperature rises.

[1] Annals of Surgery, December 1903.

